# Thai medical students’ attitudes regarding what constitutes a “good death”: a multi-center study

**DOI:** 10.1186/s12909-019-1510-5

**Published:** 2019-03-08

**Authors:** Panita Limpawattana, Varalak Srinonprasert, Manchumad Manjavong, Srivieng Pairojkul, Jarin Chindaprasirt, Sawadee Kaiyakit, Thitikorn Juntararuangtong, Kongpob Yongrattanakit, Thunchanok Kuichanuan

**Affiliations:** 1 Division of Geriatric Medicine, Department of Internal Medicine, Faculty of Medicine, Khon Kaen University, Khon Kaen, Thailand; 2 Division of Geriatric Medicine, Department of Internal Medicine, Siriraj Hospital, Faculty of Medicine, Mahidol University, Bangkok, Thailand; 3 Palliative care unit, Faculty of Medicine, Khon Kaen University, Khon Kaen, Thailand; 4 Division of Oncology Medicine, Department of Internal Medicine, Faculty of Medicine, Khon Kaen University, Khon Kaen, Thailand; 5 Residency training in Department of Internal Medicine, Faculty of Medicine, Khon Kaen University, Khon Kaen, Thailand

**Keywords:** Medical education, Palliative care, Survey, Undergraduate curriculum

## Abstract

**Background:**

Few studies exist regarding the perception of medical students toward older adults’ wishes during their end-of-life period. Better understanding of students’ perceptions regarding this topic could help improve palliative education. The purposes of this study were to examine the perceptions of medical students regarding what constitutes a “good death” and to demonstrate the factors associated with the necessary care decisions in older patients.

**Methods:**

This is a cross-sectional study**.** A questionnaire was developed and given to all of the medical students at two medical schools in Thailand (Siriraj and Srinagarind Hospital) from September 2017 to February 2018. They were asked to response to the questions by imagining how older people would think, and their preferences regarding care at the end-of-life period. The anonymous questionnaires were collected and analyzed.

**Results:**

A total of 1029 out of 2990 surveys were returned (34.4%). A minority of the sixth-year medical students rated themselves as being knowledgeable about palliative care (11.3%). According to the survey, desire to have spiritual needs met and have their loved ones present were the most important conditions that contributed to a “good death”. Factors associated with reluctance to receive prolonged treatment were female sex (adjusted odds ratio (AOR 1.39), being in the clinical years of training (AOR 1.92), self-rated good health (AOR 1.45), and prior experience of watching someone dying (AOR 1.61). Enrollment in Srinagarind medical school (AOR 2.05), being a clinical student (AOR1.91), and being dissatisfied with life (AOR 1.78) were independent factors related to preference for home death.

**Conclusions:**

Most medical students signified understanding of concepts of geriatric palliative care but felt that they had insufficient knowledge in this area. Multiple factors related to decision regarding the care that was required were identified. Medical schools should consider this information to improve geriatric palliative medical education in undergraduate training.

## Background

As people tend to have greater life expectancies than in the past due to the advances in modern medicine, the growth of the geriatric population has become a global public health challenge. Most adults develop at least one chronic disease that they may have for the rest of their lives [1, 2]. The most common chronic illnesses are those that result from and cardio-metabolic abnormalities and degenerative changes such as diabetes mellitus, hypertension, chronic renal failure, stroke, coronary artery disease, cancer and dementia. These types of chronic illnesses can lead to developing delirium in older patients and contribute to independency or incapacity of patients in making decisions toward their end-of-life period [3] Studies in older adults in the most advanced stages of illness have found that those patients often receive insufficient management of physical and psychological distress due to the complexity of their diseases [2, 4]. Geriatric palliative care is more complicated than adult palliative care because the nature and duration of their chronic illness differs from those illnesses from which younger patients suffer. Geriatric palliative care involves not only treating principal disease process but also managing several chronic medical comorbidities and geriatric syndromes such as delirium and frailty. Moreover, a significant proportion of palliative care in older people occurred in patients with advance stage non-cancerous illnesses; the unpredictable nature of their prognosis lead to difficulty in establishing goal of care. Furthermore, caregivers’ needs for older people differ to those of the caregivers of the younger patients, partly from having more symptoms requiring adequate controlling among older patients [2, 5]. This makes achieving a genuine “good death” in older adults in their end-of-life stage challenging for healthcare providers. Previous review showed that the major concerns of a good death included pain and symptom management, preparation of death, achieving a sense of completion, decisions about treatment preferences and being treated as a “whole person” [6].

Medical students are the future healthcare professionals who will take care of older adults with incurable illnesses. Care for these patients requires that students receive a holistic education, which includes not only medical knowledge and skills but also emphasizes their attitudes [7]. Perception of a good death among these students might vary according to personal experience and sociocultural background [7–9]. There has been relatively more research about what constitutes a good death from the perspective of patients and families than from that of healthcare providers, particularly medical students [7, 10–12]. One report among Thai medical students in their final year found that they perceived themselves to be proficient in holistic care and communication skills, but lacking in the ability to manage common symptoms and with regard to the ethical aspects of this kind of care [11, 13]. A study conducted among first and fifth-year German medical students found that being free from pain and physical distress was only one significant element of a good death [7]. Third-year medical students in the US and medical students in their final year in Canada perceived patient deaths as strongly emotional for students and required additional support from their supervisors in dealing with these kinds of situations [10, 14]. To our knowledge, there has been little research conducted about Thai medical students’ beliefs as to what constitutes a “good death” particularly in viewpoint for older adults. There is a lack of evidence whether the traditional Buddhist’ beliefs in Thai culture influence the perception of medical students in this regard. The examples of belief around dying in Thai people are family members have to pay back a “debt of life” to their elders by providing high-tech hospital care even the elders come to the terminal stage of their conditions, elders should not know about the whole truth about their illnesses as it can hurt their feeling and lead to deterioration of their conditions, and home is the ethical location of death in their views [8, 9]. A better understanding of medical students’ attitudes with respect to palliative care would be useful in devising an undergraduate curriculum aimed at instilling competency in this area. One way of learning about their knowledges and perspectives is to explore how they understand patients’ wishes. Therefore, the aim of this study is to examine what medical students feel constitutes a “good death” in older adults. In addition, this study aims to identify factors associated with medical students’ understanding of elderly adults’ wishes with regard to place of death and whether or not to prolong life if the chances of survival are slim.

## Methods

### Participants and settings

This cross-sectional study was a multi-center study that included first to sixth-year medical students from two medical schools in Thailand (Siriraj Hospital at Mahidol University in Bangkok and Srinagarind Hospital at Khon Kaen University in Khon Kaen) from September 2017 to February 2018. Bangkok is located in central Thailand, and Khon Kaen is located in the northeast.

Thailand is in an early stage of palliative care education development, and there is currently no standardized core curriculum. This means that the courses taught differ among medical schools [11]. It is not a compulsory course at either Siriraj or Srinagarind hospital. At Srinagarind hospital, lectures regarding palliative care education are conducted during students’ first and fourth years of training. During their clinical years, the concept of palliative care is integrated in the form of complex cases that students encounter during their rotation in the internal medicine department. For Siriraj hospital, palliative care was introduced during students’ second through fourth years of training as conventional and interactive lectures. Students were exposed to another case discussion in their sixth year during their internal medicine rotation.

### Instrument

The questionnaire was developed based on a literature review of desired element of a “good death”. The details of the questionnaire’s development were described in previous study [15]. In brief, the questionnaire was developed based on literature review of studies from Eastern and Western countries. Items selection and modification was conducted aimed to achieve the questions compatible with local culture. During the process of planning for the current study, the questionnaire was distributed to authors in the present study to review the content whether this would be suitable for culture and practice in Thailand. Authors in original study [15] and present studies comprise several geriatricians and multidiscipline team in the area of palliative care in Thailand. The questionnaire consisted of 14 main questions about demographic data, health status, previous experience in end-of-life care, and opinions toward various aspects of the end-of-life period including physical and psychological needs, autonomy issues, and closure of life affairs. The present instrument regarding students’ opinions on these topics consisted of 13 items to be rated on a five-category Likert-type scale. The participants were asked to imagine should they were older patients who were reaching the last 3 months of their lives and to rate how much they agree with the statements on the questionnaire. The participants were also asked to rank what they thought were the three most important aspects of palliative care for those older people.

Approval of research protocol from Siriraj hospital and the Khon Kaen University Institutional Review Board were obtained (reference number Si 621/2017 and HE 601309, respectively). The Khon Kaen University Ethics Committee determined that the project could be exempted for review since it was relevant to type of research according to KKU’s announcement no. 1877/2559 that involved survey procedures, interview procedures or observation of public behavior. The requirement for informed consent was, thus, waived at Khon Kaen University. The Siriraj Institutional Review Board considered this study as an expedited category and has approved for the final protocol where participants were informed of the study but written consents were not required at Siriraj hospital. This study was reported according to the STROBE guidelines [16].

### Procedure

A paper or electronic questionnaire was distributed to all medical students at Siriraj and Srinagarind Hospitals. The participants were asked to complete the questionnaire on their own convenience. Anonymity was assured, and no incentives were offered. The completed questionnaires were then sent back to the researchers.

### Statistical analysis

Demographic data were analyzed using descriptive statistics and presented as percentage, mean, and standard deviation. If the distribution of these data did not conform to normal distribution, medians and inter-quartile ranges were used instead. For factors associated with the relevant items (the decision not to prolong treatment and place of death), responses that indicated agreement with the statements were collapsed into two categories (totally agree and agree), while the rest were classified as disagreeing. Stepwise backward multiple regression was used to analyze the outcomes. A *p*-value < 0.05 was considered to indicate statistically significant differences. Adjusted odds ratios (OR) and their 95% confidence intervals (CI) were reported to denote the strength of association. The missing data were a kind of missing completely at random (MCAR) which it was safe to remove these data. The missing data were analyzed as missing. All data analysis was carried out using STATA version10.0 (StataCorp, College Station, Texas).

## Results

### Characteristics of participants and their responses regarding older adults’ wishes when it comes to end-of-life care

A total of 1029 out of 2990 of the questionnaires were completed and returned (34.4%; 1400 from Siriraj hospital and 1590 from Srinagarind hospital). The baseline demographic data of the participants are shown in Table 1. The majority of them were Buddhist, and their median age was 21 years. The highest number of respondents were from the fourth-year medical students (25%). Nearly half of the participants lived with older adults, and most of them rated themselves as being in good health and satisfied with their lives. Less than 10% of them considered themselves knowledgeable regarding palliative and end-of-life care.

**Table 1:** Baseline characteristics of the study population

Characteristics	*N* = 1029
Medical school, n(%)	
- Siriraj hospital	575 (55.9)
- Srinagarind hospital	454 (44.1)
Age (years), median (IQR1,3)	21 (20,22)
Gender^a^, n(%)	
- Female	497 (48.4)
- Male	529 (51.6)
Religion^a^ (%)	
- Buddhism	973 (94.8)
- Christianity	27 (2.6)
- Islam	2 (0.2)
- Others	16 (1.6)
- None	8 (0.8)
Year of medical student (%)	
- First	139 (13.5)
- Second	232 (22.6)
- Third	130 (12.6)
- Fourth	258 (25.1)
- Fifth	199 (19.3)
- Sixth	71 (6.9)
Family size (person)^a^; n (%)	
- Less than 3	155 (15.1)
- Three or more	871 (84.9)
Experience living with an older adult^a^, n(%)	501 (48.8)
History of hospital admission^b^, n (%)	454 (44.3)
In good health^c^, n (%)	685 (66.9)
Satisfaction in life^c^, n (%)	846 (82.5)
Prior experience of watching someone dying ^c^, n (%)	756 (73.6)
Prior experience of caring for someone at the end of their life^c^, n (%)	446 (43.4)
Prior experience counselling a patient/family member regarding end-of-life care^c^, n (%)	159 (15.5)
Self-rated as being knowledgeable with regard to palliative and end-of-life care^c^, n (%)	75 (7.3)
- First	2 (1.4)
- Second	16 (6.9)
- Third	8 (6.2)
- Fourth	22 (8.6)
- Fifth	19 (9.6)
- Sixth	8 (11.3)

Note: ^a^; 3 missing data, ^b^; 4 missing data, ^c^; 5 missing data, ^c^; 2 missing data

Participants’ responses to the questionnaire are shown in Table 2. The most important end-of-life wish reported by preclinical students (501 participants; 48.7%) was to be surrounded by their loved ones (31%), followed by being respected, having their spiritual needs met in addition to disease treatment (16.5%), completing unfinished business, being prepared to die, and saying goodbye to family and friends (13.2%). Similar results were found in clinical students (528 participants; 51.3%), with being surrounded by loved ones reported as being most important (20.1%), followed by receiving the full truth about their illnesses (16.9%), and relief of distressing symptoms (13.2%). With regard to expectation of older patients’ wishes, More than 80% of the participants “strongly agreed” that these patients wanted to be respected, have their spiritual needs met in addition to disease treatment, and to be surrounded by their loved ones in their time of need. In contrast, about a quarter to a third of the respondents answered “neutral,” “disagree,” or “strongly disagree” when asked whether patients at the end of life would prefer to know all the truth about their illnesses, not to receive treatment to prolong life when their chances of surviving are slim, to have religious rites conducted at the end of their lives, to be mentally aware in the last hour of life, and to pass away at home.

**Table 2:** Participants’ responses to questions regarding what older patients were likely to want during end-of-life care

Statements	Rating: no. of participants				
	5 Strongly agree	4 Agree	3 Neutral	2 Disagree	1 Strongly disagree
1. Patients want to know the full truth about their illnesses^a^	484 (47.4%)	456 (44.6%)	72 (7.1%)	6 (0.6%)	3 (0.3%)
2. Patients want their family to know the full truth about their illnesses^a^	299 (29.3%)	413 (40.4%)	245 (24.0%)	60 (5.9%)	4 (0.4%)
3. Patients want to be involved in the decision about the treatment they receive^b^	664 (65.3%)	307 (30.2%)	41 (4.0%)	3 (0.3%)	2 (0.2%)
4. Patients want to name a surrogate decision maker regarding their healthcare in advance for when they are unable to make these kinds of decisions for themselves^c^	483 (47.4%)	400 (39.2%)	113 (11.1%)	17 (1.7%)	6 (0.6%)
5. Patients want as much relief as possible from uncomfortable symptoms such as pain and shortness of breath ^a^	668 (65.4%)	282 (27.6%)	65 (6.4%)	3 (0.3%)	3 (0.3%)
6. Patients wish to be respected and have their spiritual needs met in addition to being treated for their diseases^a^	847 (83.0%)	152 (14.9%)	18 (1.7%)	1 (0.1%)	3 (0.3%)
7. Patients wish to be surrounded by their loved ones in their times of need^a^	817 (80.0%)	176 (17.2%)	23 (2.3%)	1 (0.1%)	4 (0.4%)
8. Patients do not wish to be a physical and psychological burden to their family^c^	580 (56.9%)	319 (31.3%)	100 (9.8%)	17 (1.7%)	3 (0.3%)
9. Patients wish to complete unfinished business, be prepared to die, and say goodbye to family and friends^d^	665 (65.2%)	296 (29.0%)	54 (5.3%)	3 (0.3%)	2 (0.2%)
10. Patients do not wish to receive treatments to prolong their lives when the chances of surviving are slim^d^	338 (33.1%)	382 (37.5%)	244 (23.9%)	44 (4.3%)	12 (1.2%)
11. Patients wish to have religious rites conducted at the end of life^d^	409 (40.1%)	344 (33.7%)	243 (23.8%)	20 (2.0%)	4 (0.4%)
12. Patients wish to be mentally aware during the last hour of life^c^	386 (37.9%)	353 (34.6%)	249 (24.4%)	23 (2.3%)	8 (0.8%)
13. Patients wish to pass away at home^a^	383 (37.5%)	306 (30.0%)	300 (29.4%)	21 (2.0%)	11 (1.1%)

Note: ^a^; 8 missing data, ^b^; 12 missing data, ^c^; 10 missing data, ^d^; 9 missing data

### Factors associated with medical students’ not wanting to receive life-prolonging treatment when the chances of survival are slim and desire for a home death

According to stepwise regression analysis, female sex, being in the clinical years of training, rating oneself as being in good health, and having had prior experience caring for someone during their end-of-life period were independent factors associated with not wanting to receive life-prolonging treatment when the chances of survival are slim (Table 3). Being at Khon Kaen university, being in the clinical year of training, and being dissatisfied with life were significant factors associated with wishing to pass away at home (Table 4).

**Table 3:** Factors associated with participants answering that elderly patients would not wish not to receive life-prolonging treatment when the chances of surviving are slim according to stepwise regression analysis

Factors	Adjusted OR	(95% CI)	*p*-value
Female	1.39	(1.05,1.84)	0.02*
Clinical year of training	1.92	(1.40,2.66)	0.00*
Experience living with older adult	1.20	(0.91,1.59)	0.19
In good health	1.45	(1.09,1.95)	0.01*
Prior experience of watching someone dying	1.61	(1.13,2.31)	0.008*
Prior experience of caring for someone at the end of their life (%)	0.80	(0.57,1.12)	0.19

*: *p*-value < 0.05

**Table 4:** Factors associated with participants answering that elderly patients would prefer to pass away at home using stepwise regression analysis

Factors	Adjusted OR	(95% CI)	*p*-value
Medical school			
- Siriraj hospital	1	–	–
- Srinagarind hospital	2.05	(1.55,2.72)	0.00*
Clinical year of training	1.91	(1.41,2.58)	0.00*
Not satisfied with life	1.78	(1.20,2.63)	0.004*
Prior experience of watching someone dying	1.24	(0.89,1.73)	0.20

*: *p*-value < 0.05

## Discussion

The results of this survey reflect some perspectives of Thai medical students regarding geriatric palliative care in the central and northeast regions of Thailand. These students are the nation’s future healthcare providers, and their opinions might partly reflect the role of Buddhism in Thai society. Several reports from around the world show that medical students feel unprepared to provide palliative and end-of-life care, in terms of both knowledge and practice, even if they had been receiving education in palliative care since they were preclinical students [13, 17, 18]. This reported uneven and fragmented training, is consistent with the results of a previous study in Thailand that found that the majority of students (even those in their sixth year) were not confident in providing palliative care services by themselves [11, 13]. Only about 11% of the sixth-year students in this study rated themselves as being knowledgeable about palliative care, whereas another published study in Thailand reported at least 80% of sixth-year students were confident that they could manage the holistic care and communication aspects of palliative care but not ethical aspects or symptom management [11]. This discrepancy might be explained by the differences in study settings, structure of palliative care education of each medical school, and details of the questionnaires. Therefore, direct comparison might be difficult. The possible reasons of low self-rated as being knowledgeable about palliative and end-of-life care in this study were the majority of them had no experience in living with an older person, caring for someone at the end of life period, and most of them were in the first- to fourth-year of training where experience in real clinical practice is less than the fifth-and sixth-year medical students.

In this study, it appears that the majority of respondents believed that older patients concern about all domains involved in comprehensive palliative assessment including their physical and psychological needs, autonomy, and closure of life affairs. Interestingly, they placed the highest rank of their focus on the desire for spiritual needs to be met and to be surrounded by their love ones. It is also noteworthy that other questions regarding spiritual needs such as surrounded by love ones, wish to prepare for the death [19] were also rated by high proportion of respondents. There has been limited studies focusing on medical students regarding spiritual needs for end of life patients [20]. The finding implies that medical students signify the importance of spiritual needs and psychosocial health which should be emphasized and sustained through the training curriculum for medical students.

Another interesting finding was the high proportion of medical students addressed the important of having patients involved in their decision making. This suggests that members of the younger generation in Asian countries tend to value autonomy in medical decision making. This is a point of view that contrasts with traditional Chinese, Japanese, and Korean practices. In those practices, decision-making tends to be family and physician-based rather than to patient-based [12, 21], there is an attempt to protect the patient by not informing of them of their prognosis [22], and a “managed death” is more important than a “good death” [7, 23]. For example, the perspective view of Chinese culture about death and dying are taboo issues that are inappropriate for open discussion and family seems to be a basic social component for making decision about care plan [24]. Japanese traditional concepts of death signify about unawareness of death. Good death in their view is living as usual without the feeling of facing impending death [25]. Korean-American also more favored a family-centered model for medical decision making than the patient autonomy which commonly found in African-American and European-American [26]. The findings from the present also corresponded to those of a previous study that examined the end-of-life preferences among elderly Thai residents who preferred to be informed of prognosis and be involved in making decision [15].

The significant factors associated with unwillingness to receive life-prolonging treatment when chances of survival are low were female sex, being in clinical training, rating oneself as being in good health, and prior experience of watching someone dying. Gender differences have been mentioned in other studies that examined perceptions of a good death, which found that women tended to focus on well-being as a core component of health, where men tended to emphasize autonomy and independence [7, 27, 28]. Medical students in their preclinical years focus mainly on developing their knowledge regarding end-of-life care rather than their skills or attitudes. Clinical students who had had face-to-face experience with the patients in real situations were, thus, likely to have a better understanding of palliative care and suffering patients could receive. Medical students with prior experience of watching someone dying also had positive attitudes about palliative care. This finding was consistent with those of a prior report that found that the clinical experience with patients at the ends of their lives changed students’ attitudes with regard to dealing with dying patients [29].

Place of death is another important concern for terminally ill patients, and there have been a number of reports regarding discrepancy of opinion on this issue between the patient and their family or the patient and their healthcare providers [9, 12, 30]. This discrepancy influences the aggressive management at the end-of-life period [12, 31]. Being a student at Khon Kaen University, being in the clinical years of training, and dissatisfaction in life were significant factors related to students’ assuming that elderly patients preferred to die at home according to multivariate regression analysis. A significantly higher percentage of medical students from Khon Kaen University (in northeast Thailand) expected that dying older patients preferred to die at home than those studying at Siriraj hospital in central part of Thailand. Ethnographic data could be a potential explanation for this difference. Native individuals are likely to feel that it is ethical to withdraw life support at home but unethical to do so in a hospital [9]. The traditional beliefs of the residents of this region are shaped by their Buddhist faith, in which home death is associated with a better rebirth. In addition, they tend to associate a “good death” with being under the care of their family, neighbors, and friends, while hospitals are a place for saving lives. Hospitalized patients at the end of their lives are often transported to their home by ambulance to withdraw life support due to these ethical considerations [8, 9]. However, living in urban areas, such as Bangkok, makes palliative care at home more challenging due to factors such as the low level of support from the public health system for home palliative care and a weaker family network [32]. The preferences of residents of urban areas have shifted toward preferring to die in a hospital, as demonstrated by a previous survey regarding the wishes of elderly patients in Bangkok [15]. Being in their clinical years of training also have them agreed more with the preference to die at home. This could, again, be explained by the fact that clinical students had had more experience with real-world patients and the incorporation of traditional beliefs into their care, making their ideas about the preferences of elderly patients tended to coincide with those of the general population [9]. A similar result was found in a study conducted in German medical students, approximately half of whom (43.5%) preferred home death to dying in other places [7]. Self-rated dissatisfaction in life was another related factor for all medical students related to the preference to die at home. A probable explanation for this is that, according to traditional Thai beliefs, the home is a sacred place, and being in a familiar surrounding will bring about a peaceful death [8]. Thus, those with low life satisfaction tend to prefer to pass away in the home.

The findings from study suggest that medical education with respect to geriatric palliative-care could be improved in a variety of ways such as providing knowledge about opinion about good death from older person’s perspective, particularly on involving them to make decision making and prefer place of death. These could be achieved through good communication. Therefore, the promotion of communication skills, the implementation of palliative care into compulsory training particularly on their clinical years would be some important aspects to be addressed. Moreover, having more hand-on experience under supervision using standardized patient and using group sessions for reflection might be a good leaning model. For example, palliative care is implemented as a mandatory cross-disciplinary subject at the Medical Faculty of the Heinrich-Heine-University Düsseldorf, Germany. They have a variety of teaching programs for palliative care educator such as video, e-learning module, interprofessional education, group sessions for reflective self-development. The results of this process were favorably valued by medical students, participating patients and their families [33]. Additionally, the development of a formal evaluation process in real clinical practice would also be advisable.

This study had several limitations. First, although the number of participants was high, but a response rate was 34% and conducted in 2 regions out of 5 in Thailand. These results might not be generalizable to all medical students in Thailand, as palliative care curricula might be integrated in different ways in various institutes. Nevertheless, these 2 university hospitals have established palliative care service and education for more than 10 years, the feature of low confident regarding palliative care among students in the study is even more concerning for the whole country. Secondly, the study about wishes of Thai older adults was conducted only in Bangkok [15]. The regional/cultural backgrounds of participants could also affect their preferences. Further studies should be conducted in other parts of Thailand and directly compare the attitudes of medical students or other healthcare professionals with those of older adults. Such research would aid in the development of better care strategies and facilitate patients’ ability to have a “good death.” Lastly, the questionnaire was developed in a context of Buddhist culture in Thailand. Therefore, application the results in other religions might be limited. Additional research about opinions regarding wishes at the end-of-life care from Muslim and Christian aspects in the Thai context and other countries are required.

## Conclusions

This study examined the perceptions of Thai medical students (the next generation of physicians) regarding what constitutes a “good death” in a Buddhist society. The majority of them understood the concept of palliative care, but even those in their final year of training felt they lacked knowledge in geriatric palliative care. Factors related to a desire not to receive life-prolonging treatment when the chances of survival are low were female sex, being in clinical training, rating oneself as being in good health, and prior experience watching someone dying. Enrollment in Khon Kaen University’s medical school, being in clinical training, and dissatisfaction with life were significant factors related to a preference for home death. The results from this study could be used to help improve undergraduate curricula by focusing more issues on palliative care for older patients by promotion of communication skills, the implementation of geriatric palliative care as a mandatory training, using standardized patient in the study modules, and the development of a formal evaluation process in real clinical practice in order to contributing to a more efficient therapeutic practice of these future physicians. Further researches regarding direct comparison of the attitudes between medical students and those of older adults, and opinions about wishes in the context of other religions in the Thai context would benefit for improving students’ performance.

## Abbreviations

CI: Confidence interval
MCAR: Missing completely at random
OR: Odds ratio
STROBE: Strengthening the Reporting of Observational studies in Epidemiology
